# Phosphopeptidome Profiling of Human Plasma for Hepatocellular Carcinoma Biomarker Discovery

**DOI:** 10.1021/acs.jproteome.5c01004

**Published:** 2025-12-31

**Authors:** Shafaq Saleem, Muhammad Salman Sajid, Rency S. Varghese, Zaki A. Sherif, Alexander Kroemer, Habtom W. Ressom

**Affiliations:** 1 Department of Oncology, Lombardi Comprehensive Cancer Center, 12231 Georgetown University Medical Center, Washington, D.C. 20057, United States; 2 Department of Biochemistry & Molecular Biology, Howard University College of Medicine, Washington, D.C. 20059, United States; 3 MedStar Georgetown Transplant Institute, MedStar Georgetown University Hospital and the Center for Translational Transplant Medicine, 12231 Georgetown University Medical Center, Washington, DC 20057, United States

**Keywords:** liver cancer, endogenous phosphopeptides, biomarker, human plasma, nano-LC-MS/MS

## Abstract

Hepatocellular carcinoma (HCC) remains a leading cause of cancer mortality, and current biomarkers such as alpha-fetoprotein (AFP) lack diagnostic accuracy. Here, we report the first comprehensive profiling of the plasma endogenous phosphopeptidome in HCC, cirrhosis, and healthy controls using a digestion-free LC–MS/MS workflow. From 60 plasma samples, 1,365 phosphopeptides corresponding to 549 proteins were identified and quantified. Among these, the statherin-derived peptide DSSEEKFLR demonstrated outstanding discrimination between HCC and cirrhosis (AUC = 0.968), outperforming AFP (AUC = 0.648). Additional peptides, including PPGAPHTEEEGAE (NST1), YEYDELPAKDD (C4A), SLPGESEEMMEEVD (ITIH4), and VSLGSPSGEVSHPRKT (AHSG), also showed high accuracy (AUC > 0.80). Functional enrichment revealed perturbations in acute-phase response, coagulation, lipid metabolism, and LXR/RXR signaling. Collectively, this work defines a novel plasma phosphopeptide signature that reflects disease-specific proteolytic and phosphorylation dynamics, providing a foundation for developing biomarkers for early detection and clinical monitoring of HCC.

## Graphical abstract

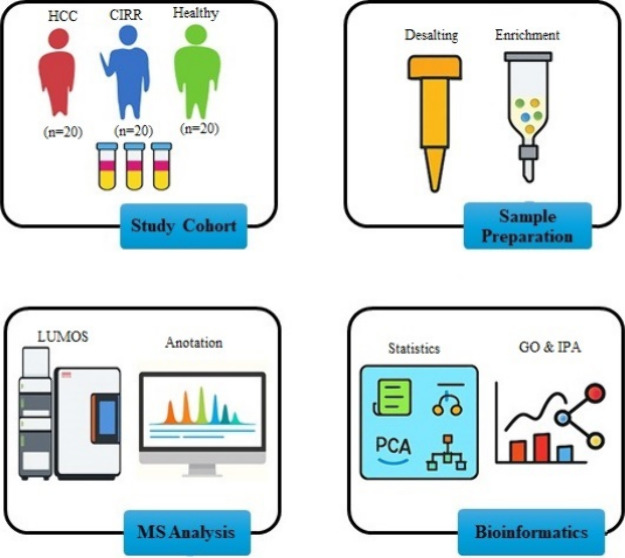

## Introduction

Hepatocellular carcinoma (HCC) is a common primary liver cancer and an alarming global public health concern.^1^ More than 900,000 new cases are detected each year, according to GLOBOCAN 2020.^2^ About 830,000 deaths happen yearly, making HCC the sixth utmost commonly diagnosed cancer and the third top origin of cancer-related deceases globally.^3^ The occurrence of this malignancy has been growing gradually in both developing and developed countries, as a result of various risk issues. Chronic viral infections caused by HBV and HCV remain to account for an extensive part of cases, particularly in areas with high endemicity.^4^ Moreover, metabolic and lifestyle factors as well as obesity, consumption of alcohol, insulin resistance, nonalcoholic fatty liver disease, and type 2 diabetes contribute pointedly to the worldwide disease burden.^5–7^

Current HCC monitoring guidelines primarily recommend abdominal ultrasound imaging, every six months in combination with serum alpha-fetoprotein (AFP) testing for individuals at high risk, typically those with cirrhosis or chronic hepatitis B or C.^8^ However, these methods have important drawbacks. In noncancer liver conditions like chronic hepatitis or cirrhosis, AFP levels can be high making it hard to rely on this test alone^9^ and ultrasounds can miss early signs of HCC about 47% of the time.^10^ Markers such as des-γ-carboxy prothrombin (DCP),^11^ and AFP-L3, combined with other factors including sex, age, and AFP perform better than alone AFP,^12–14^ but across diverse patient populations none of them provide the reliability required for early detection.^15^ Therefore, there is a pressing need to find new, noninvasive markers that accurately detect HCC in patients with liver cirrhosis (CIRR), because liver cirrhosis often occurs alongside it and makes diagnosis tougher.

Plasma, which serves as an accessible “liquid biopsy,” offers insight into both tissue-specific alterations and overall physiological status, that makes it valuable resource for discovery of biomarker.^16^ Nonetheless, studying the plasma proteome remains technically challenging for its wide dynamic range and the dominance of a few highly abundant proteins that can obscure the detection of low-abundance biomarkers.^17^ Recent technological advancements, such as high-resolution mass spectrometry, enhanced chromatographic separation techniques, and robust quantitative workflows, have helped a more detailed investigation of the plasma proteome.^18^ Furthermore, multimarker panels identified from proteomic analyses have demonstrated better performance compared to AFP alone in distinguishing HCC from chronic or cirrhosis hepatitis.^19^ In addition to conventional proteomic workflows based on tryptic digests, body fluids inherently contain low molecular weight peptides collectively referred to as the endogenous peptidome.^20^ These endogenous peptides arise from protein secretion, proteolytic processing, or protein degradation, and considered to deliver “snapshots” of pathological or physiological states.^21^ Endogenous peptides offer several advantages as biomarkers: they are normally stable, can be measured directly without enzymatic digestion, and frequently reflect disease associated protease activity.^22^

Recent research on profiling of endogenous peptidome in serum has detected several significantly altered peptides in HCC vs CIRR. Particularly, a peptide derived from immunoglobulin heavy constant gamma 4 exhibited perfect discrimination between HCC from CIRR in a discovery cohort.^23^ Phosphoproteomic studies in HCC have also reported dysregulated phosphorylation pathways in tumor tissues and experimental models, highlighting alterations in cell-cycle control, metabolic regulation, and kinase.^24,25^ While these studies provide insight into intracellular signaling changes, they are primarily tissue-based or model-based and do not capture circulating phosphorylated peptides. Profiling the endogenous phosphopeptidome may therefore offer a minimally invasive means of detecting systemic phosphorylation and proteolytic changes associated with HCC. The endogenous phosphopeptidome, phosphorylated peptides naturally existing in circulation remains largely unexplored. Profiling the endogenous phosphopeptidome could offer the combined advantages of both approaches: the accessibility of endogenous peptides and the biological specificity conferred by phosphorylation. This provides a powerful and novel window for exploratory systemic signaling alterations associated with HCC. However, the endogenous phosphopeptidome poses significant technical challenges. The abundance of phosphorylated endogenous peptides is predictable to be even lower than that of nonphosphorylated peptides, demanding highly sensitive enrichment strategies and high-resolution detection methods.

Herein, we report profiling of human plasma endogenous phosphopeptidome in a cohort of 60 patients with HCC (*n* = 20), CIRR (*n* = 20), and healthy controls (CTL) (*n* = 20) to identify HCC candidate biomarkers. Endogenous peptides were released from yellow plasma, and enriched for phosphopeptides prior to analysis on a nanoLC-Orbitrap LUMOS mass spectrometer. Label-free quantification followed by statistical evaluation using one-way ANOVA identified the significant alterations across the three groups. Multivariate analysis revealed distinct clustering of HCC from CIRR and CTL, while functional annotation highlighted pathways relevant to liver disease progression and tumor biology. By integrating phosphopeptidome profiling with quantitative and bioinformatic analyses, this work provides the first systematic characterization of circulating endogenous phosphopeptides in HCC and establishes their potential as a novel reservoir of minimally invasive candidate biomarkers for HCC.

## Materials and Methods

### Experimental Design

The overall experimental workflow for this study is summarized in Figure 1. Plasma samples obtained from HCC, CIRR, and CTL were processed under acidic condition to release endogenous peptides followed by desalting and enrichment of released phosphopeptides. The samples were then analyzed on a nano-LC coupled with Orbitrap Fusion Lumos Tribrid mass spectrometer (Thermo Fisher Scientific, Waltham, MA, USA). Data were processed in Proteome Discoverer (PD) for annotation and label-free quantification. Rigorous quality control was performed using HeLa digest standards injected after each batch of HCC, CIRR, and CTL plasma samples. For each QC run, 200 ng of HeLa digest was analyzed under identical conditions on the mass spectrometer. Statistical and bioinformatics analyses of the processed LC-MS/MS data included ANOVA with Tukey’s post hoc tests, chi-squared testing for presence/absence patterns, and Gene Ontology (GO) enrichment and pathway mapping, as well as protein–protein interaction (PPI) network analysis using STRING and Ingenuity Pathway Analysis (IPA).

**Figure 1:**
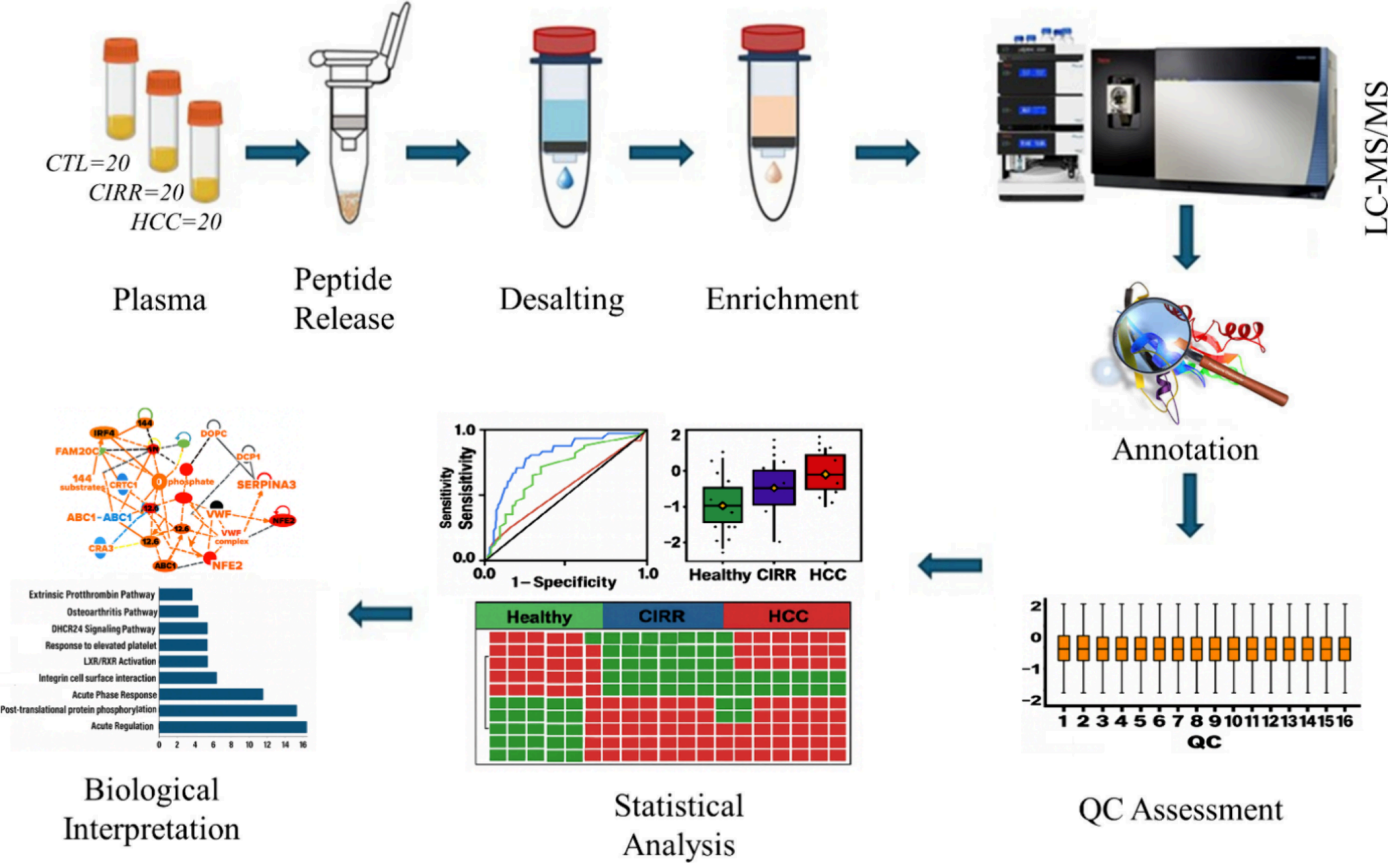
Workflow of plasma endogenous phosphopeptidome profiling for HCC biomarker discovery.

### Study Cohort

Plasma samples from 60 subjects, comprising 20 patients with HCC, 20 patients with liver cirrhosis, and 20 healthy volunteers were selected for this study from our repository in such a way that the three groups do not differ significantly in gender, age, or other clinical characteristics. Also, none of the subjects had alcohol-related liver disease or nonalcoholic fatty liver disease, thereby reducing potential confounding factors and enabling a clearer assessment of disease-specific molecular alterations. Detailed demographic and clinical characteristics of all subjects are summarized in Table 1. All participants were recruited from MedStar Georgetown University Hospital (MGUH) and Howard University Hospital, and the study protocol was approved by the Georgetown University Institutional Review Board (IRB). Written informed consent and Health Insurance Portability and Accountability Act (HIPAA) authorization were obtained from all participants prior to enrollment. For HCC cases, diagnoses were confirmed by diagnostic imaging and histopathological examination, with clinical staging determined according to the tumor node metastasis (TNM) classification system. Peripheral venous blood was collected using sterile vacuum tubes without anticoagulants. Samples were centrifuged at room temperature, first at 1000 × g for 10 min, followed by a second centrifugation at 2500 × g for 10 min. The resulting serum was carefully collected, aliquoted in the presence of protease inhibitors, and stored at – 80 °C until further analysis.

**Table 1:** Demographic and clinical characteristics of the study cohort (*n* = 60). Abbreviations: AA, African American; EA, European American; HCV, hepatitis C virus; HBV, hepatitis B virus; Ab+, antibody positive; Ag+, antigen positive; AFP, alpha-fetoprotein; MELD, Model for End-Stage Liver Disease

		HCC (*n* = 20)	CIRR (*n* = 20)	CTL (*n* = 20)
age	mean (SD)	59.7 (6.5)	57.7 (6.6)	49.8 (11.2)
gender	male	0.6	0.65	0.22
race	AA	0.5	0.35	1
	EA	0.5	0.65	
etiology	HCV	0.85	0.75	
	alcohol	0.35	0.4	
HCV serology	HCV Ab+	0.75	0.7	
HBV serology	anti HBC+	0.35	0.3	
	HBs Ag+	0	0	
smoking	current	0.2	0.15	0.375
	former	0.6	0.5	
	none	0.2	0.35	
alcohol	current	0.15	0.2	
	former	0.65	0.6	0.09
	none	0.2	0.2	
AFP	median (IQR)	39 (79.1)	7 (35.6)	
MELD	median (IQR)	10 (4.3)	13 (9.3)	
HCC stage	stage I	0.25		
	stage II	0.65		
	stage III	0.05		

### Materials

All reagents were analytical or LC–MS grade unless otherwise noted. Trifluoroacetic acid (TFA; Thermo Fisher Scientific, Cat. No. 85183), formic acid (FA; Thermo Fisher Scientific, Cat. No. A117–50), and acetonitrile (ACN; Thermo Fisher Scientific, Cat. No. A955–4) were used as LC–MS grade solvents, and ultrapure water was produced using a Milli-Q purification system (MilliporeSigma). Endogenous peptides were isolated using Amicon Ultra 0.5 mL centrifugal filter units (10 kDa MWCO; MilliporeSigma, Cat. No. UFC501096), desalted using Oasis HLB 1 cc Vac Cartridges (10 mg; Waters, Cat. No. WAT094226), and enriched for phosphopeptides with the Fe-NTA Phosphopeptide Enrichment Kit (Thermo Fisher Scientific, Cat. No. A32992). All microcentrifuge tubes and pipet tips were MS-compatible (Eppendorf or Thermo Fisher Scientific). HeLa Protein Digest Standard (Thermo Fisher Scientific, Cat. No. 88328) was used for routine quality control injections. NanoLC–MS/MS analyses were performed using a Dionex UltiMate 3000 RSLCnano system coupled to an Orbitrap Fusion Lumos Tribrid mass spectrometer (Thermo Fisher Scientific). Chromatographic separation was achieved using an Acclaim PepMap RSLC C18 analytical column (75 μm × 250 mm, 2 μm, 100 Å; Thermo Scientific, Cat. No. 164942) and a PepMap C18 trap column (75 μm × 20 mm, 3 μm, 100 Å; Thermo Scientific, Cat. No. 164535). Endogenous phosphopeptide identification and label-free quantification were performed using Proteome Discoverer 3.0 (Thermo Fisher Scientific). Functional enrichment analysis was conducted using Ingenuity Pathway Analysis (IPA; Qiagen), and statistical analyses, including ANOVA and PCA, were performed in R version 4.3.0.

### Sample Preparation

Endogenous peptides were extracted from 100 μL of human serum using our previously reported method with minor modifications.^26^ Samples were acidified with 500 μL of 1% trifluoroacetic acid (TFA), vortexed, and heated at 95 °C for 15 min to disrupt peptide–protein interactions. After cooling to room temperature, mixtures were filtered through preconditioned with 1% TFA Amicon Ultra-0.5 centrifugal units (10 kDa MWCO) and centrifuged at 14,000 × g for 20 min at 10 °C, followed by two washes with 100 μL of 1% TFA. Filtered peptides were desalted using Oasis HLB 1 cc Vac Cartridges (10 mg), conditioned with acetonitrile and equilibrated with 0.1% TFA in water. Peptides were loaded, washed twice with 0.1% TFA in water, and eluted with 70% acetonitrile containing 0.1% TFA, then dried under vacuum.

For endogenous phosphopeptide enrichment, dried peptides were resuspended in 100 μL of loading buffer from the Fe-NTA Phosphopeptide Enrichment Kit (Thermo Fisher Scientific). Fe-NTA spin columns were equilibrated with 200 μL of loading buffer and centrifuged at 1,000 × g for 1 min. Peptide samples (100 μL) were applied to the columns and incubated for 30 min at room temperature with gentle agitation. Columns were washed twice with 200 μL of wash buffer and once with 200 μL of water to remove unbound peptides, and captured endogenous phosphopeptides were eluted twice with 70 μL of elution buffer. Eluates endogenous phosphopeptides were dried and reconstituted with 3% ACN in 0.1% FA and peptide concentration was determined with nanodrop before nano-LC-MS/MS.

### Nano-LC-MS/MS Data Acquisition

Data were acquired using a Dionex 3000 UltiMate Nano LC system interfaced to a LUMOS terebrid mass spectrometer (Thermo Scientific, San Jose, CA, USA) equipped with Easy-Spray ESI source (Thermo Fisher Scientific). One μg of peptides from each sample was injected into the LC system for analysis. To achieve preconcentration and cleanup, peptides were first passed through a C18 Acclaim PepMap trap column (75 μm × 20 mm, 3 μm, 100 Å, Thermo Scientific) before being transferred to a C18 Acclaim PepMap RSLC column (75 μm × 250 mm, 3 μm, 100 Å, Thermo Scientific). A multistage gradient was used with a total run time of 120 min. The mobile phase A was water and 0.1% formic acid (FA), while mobile phase B was 100% acetonitrile (ACN) and 0.1% FA. The column oven temperature was maintained at 35 °C throughout the run. During the initial 5 min, the mobile phase B was held constant at 4% with a 300 nL/min flow rate. It gradually increased to 35% over the next 120 min with a flow rate of 220 nL/min. Between 125 and 133 min, mobile phase B was further increased to 95% with a flow rate of 250 nL/min, and this composition was maintained for 5 min. Finally, the mobile phase B was reduced to 4% with a flow rate of 300 nL/min and held constant until the end of the run. The separated peptides were directed to the mass spectrometer at a voltage of 2.2 kV. The full MS scan was performed with a scan range of 370–1850 *m*/*z* and the analytes were detected in the Orbitrap at a resolution of 12K. The top 3 most intense ions were selected for MS2 fragmentation in a high-energy collision dissociation (HCD) cell, with a normalized collision energy (NCE) of 28 at a resolution of 6K, with a dynamic exclusion of 30 ms.

### Endogenous Phosphopeptide Annotation

Endogenous phosphopeptide identification and label-free quantification (LFQ) was performed in Proteome Discoverer 3.0 (Thermo Scientific, Waltham, MA, USA) using Sequest HT against the human UniProt database (July 2024). The workflow included mass recalibration, Minora Feature Detector, Standard Spectrum Selector, and Percolator nodes. Precursor and fragment mass tolerances were set to 10 ppm and 0.02 Da, respectively, with no enzyme specificity and phosphorylation on serine, threonine, and tyrosine (STY) as a dynamic modification. High-confidence identifications were filtered at a 1% false discovery rate using Percolator.

### Data Analysis

Clustering within each sample group was assessed by principal component analysis (PCA) using LFQ intensities of all endogenous phosphopeptides to identify potential outliers. Peptides detected in less than 70% of at least one patient group were left aside for subsequent analysis based on absent and present calls. Abundance values for the remaining endogenous phosphopeptides were log_2_-transformed, and missing values inherent to data-dependent acquisition (DDA) shotgun proteomics were imputed using the K-nearest neighbor (KNN) feature-wise method. Data were normalized by the median to reduce inter-run variation. Following significance analysis using ANOVA, differential expression of endogenous phosphopeptides in HCC vs CIRR as well as HCC vs CTL was assessed. Endogenous phosphopeptides with a fold change >1.5 and false discovery rate (FDR) < 0.05 were considered significant. Precursor proteins of differentially expressed endogenous phosphopeptides were further analyzed for pathway and functional correlations using Ingenuity Pathway Analysis (IPA) software (Qiagen Inc., Germantown, MD, USA). Differential expression of endogenous phosphopeptides detected in fewer than 70% of the samples in each group was evaluated by assessing change in peptide occurrence in HCC vs CIRR and HCC vs CTL via Pearson’s chi-squared test.

## Results

The QC injections demonstrated high reproducibility across the entire acquisition period. The distribution of log-transformed protein abundances showed tight clustering across all runs (Figure S6), while the number of proteins identified per injection remained stable with minimal variation (Figure 2c). These findings confirm the robustness of the nano-LC-MS workflow and validate that the observed differences between groups reflect biological rather than technical variation.

**Figure 2:**
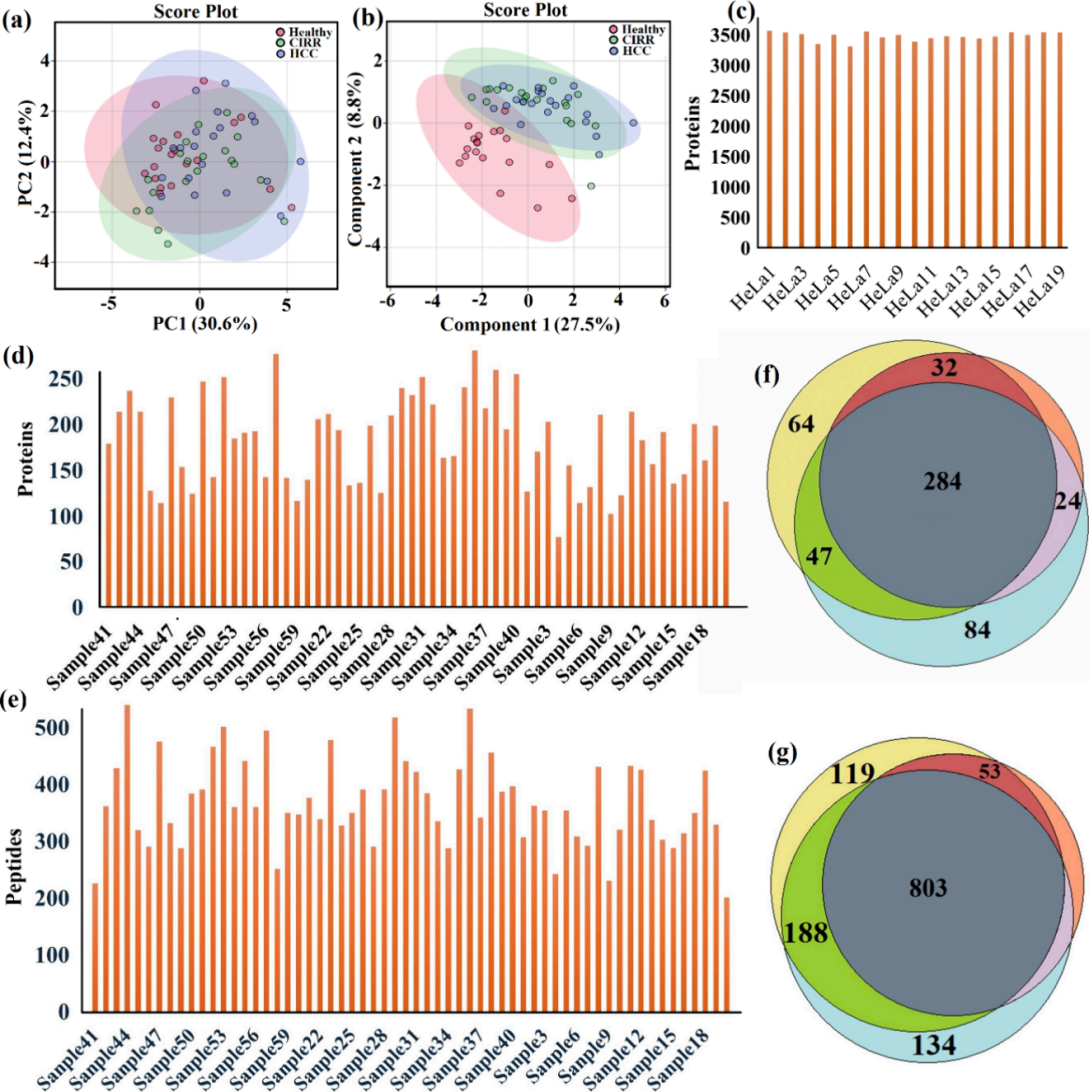
Endogenous phosphopeptide profiling of plasma samples from HCC cases, patients with CIRR, and healthy control volunteers. (a) Principal component analysis (PCA) score plot; (b) partial least-squares-discriminant analysis (PLS-DA) plot of the three groups; (c) quality control (QC) analysis using HeLa digest standards demonstrated stable MS performance across all runs; (d, e) bar plots showing the number of phosphopeptides identified in individual plasma samples across the three groups; and (f) Venn diagram depicting the overlap of precursor phosphoproteins identified among three groups. (g) Venn diagram showing the overlap at the peptide level, with 803 peptides commonly detected across all groups and subsets uniquely enriched in each condition.

Comprehensive profiling revealed a rich plasma phosphopeptidome across the 60 samples. In total, 1,365 unique endogenous phosphopeptides corresponding to 549 precursor phosphoproteins were identified (Supplementary Table S2). Correlation analysis across the 60 plasma samples demonstrated high reproducibility and internal consistency of the data set. Pairwise Pearson correlation coefficients for normalized peptide intensities were consistently high (*r* > 0.9 for most comparisons), confirming that the endogenous phosphopeptidome was measured with excellent technical stability across all runs (Figure S1). Group-wise abundance analysis further illustrated the global behavior of the endogenous phosphopeptidome. At the protein level, phosphoprotein intensities were broadly distributed across all three groups, but distinct abundance shifts were observed in disease cohorts compared with CTL (Figure S5). At the peptide level, overall phosphopeptide abundance profiles showed similar trends (Figure S4). Box plots revealed that HCC samples contained a greater number of high-intensity peptides compared with CIRR and CTL groups, as shown in Figure S3 by the most individual points (outliers) above the upper whisker. Partial least-squares–discriminant analysis (PLS-DA) confirmed distinct clustering of the diseased group from heathy controls (Figure 2b).

Consistency of phosphopeptide and precursor protein identification was demonstrated by bar plots showing little variation in counts per sample (Figure 2d–e). At the peptide level, 803 phosphopeptides were shared across all groups, while subsets were uniquely enriched: 40 in HCC, 119 in CIRR, and 134 in CTL subjects, with 188 shared between HCC and CTL and 53 shared between HCC and CIRR (Figure 2g). At the protein level, 284 phosphoproteins were common to all groups, while 84 were unique to CTL, 64 to CIRR, and 14 to HCC (Figure 2f). The identified phosphoproteins exhibited a broad distribution of physicochemical properties (Figure S2). Sequence coverage values ranged from low to moderate, reflecting partial but consistent coverage across the plasma proteome. The molecular weight (MW) distribution spanned a wide range, with most proteins detected between ∼ 10–100 kDa. The isoelectric point (pI) profile was evenly distributed across acidic, neutral, and basic proteins. At the peptide level, the endogenous phosphopeptides demonstrated wide variation in molecular features (Figure S3). The charge state distribution was dominated by doubly and triply charged peptides. The retention time (RT) distribution showed uniform peptide elution across the chromatographic gradient. The monoisotopic mass (MH^+^) values were broadly distributed, and the peptide length distribution ranged from short motifs (∼7–10 residues) to longer sequences (>20 residues), capturing the heterogeneity of circulating phosphopeptides. These features confirm that the data set captures a representative and chemically diverse view of the circulating phosphopeptidome.

To focus on biologically robust signals, peptides were filtered for 70% presence within each group 421 peptides were retained for differential expression analysis. One-way ANOVA identified 69 significantly altered peptides across HCC, CIRR, and CTL groups (FDR < 0.05, Supplementary Table S1 and Figure S7). Representative dot plots of significantly upward and downward trend when comparing all the three groups are shown in Figure 3.

**Figure 3:**
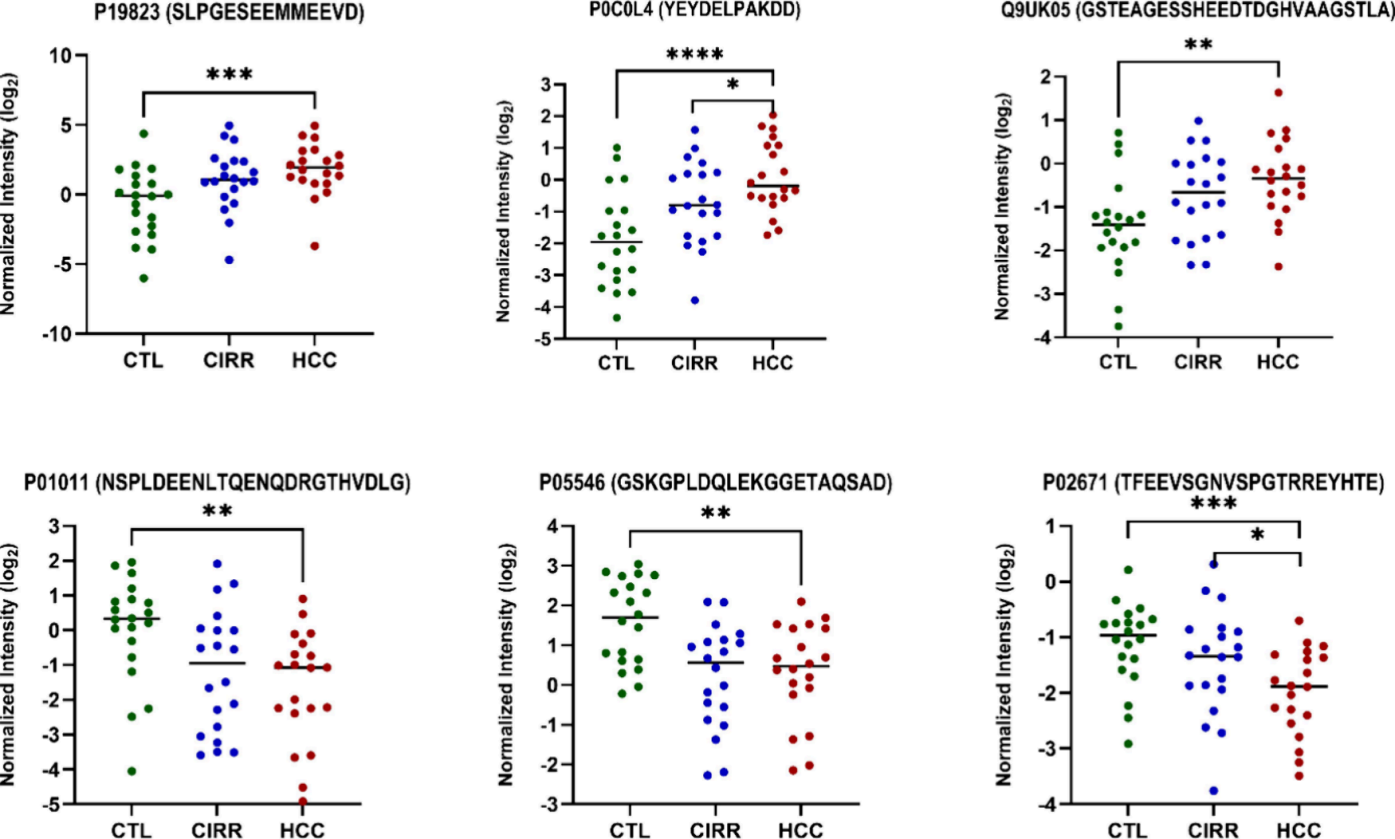
Dot plots for endogenous phosphopeptides showing upward and downward trends when comparing CTL, CIRR, and HCC together (***p < 0.0001, **p < 0.001, *p < 0.05).

Pairwise comparisons provided further insight. In the HCC vs CIRR, statherin (P02808, DSSEEKFLR & DSSEEKFL) displayed the strongest upregulation in HCC (fold change 5.26 and 3.16), while ApoA1 peptides such as GHKEVTKEVVTS showed fold changes greater than 5. VSLGSPSGEVSHPRKT (P02765) was also significantly higher in HCC (fold change 2.0). In the HCC vs CTL comparison, several phosphopeptides were significantly elevated. These included SLPGESEEMMEEVD from *Interalpha-trypsin inhibitor heavy chain* (FC = 2.9) and YEYDELPAKDD from *Complement C4-A* (FC = 3.0), both involved in inflammatory and acute-phase pathways. Conversely, multiple peptides showed decreased abundance in HCC, such as NSPLDEENLTQENQDRGTHVDLG from *Alpha-1-antichymotrypsin* (FC = – 2.8), TFEEVSGNVSPGTRREYHTE from *Fibrinogen alpha chain* (FC = – 1.8), and GSKGPLDQLEKGGETAQSAD from *Heparin cofactor 2* (FC = – 2.3).

Representative dot plots of significantly upregulated and downregulated peptides are shown in Figures 3 (HCC, CIRR, and CTL) and 4 (HCC vs CIRR), with complete results presented in Table 2.

**Figure 4:**
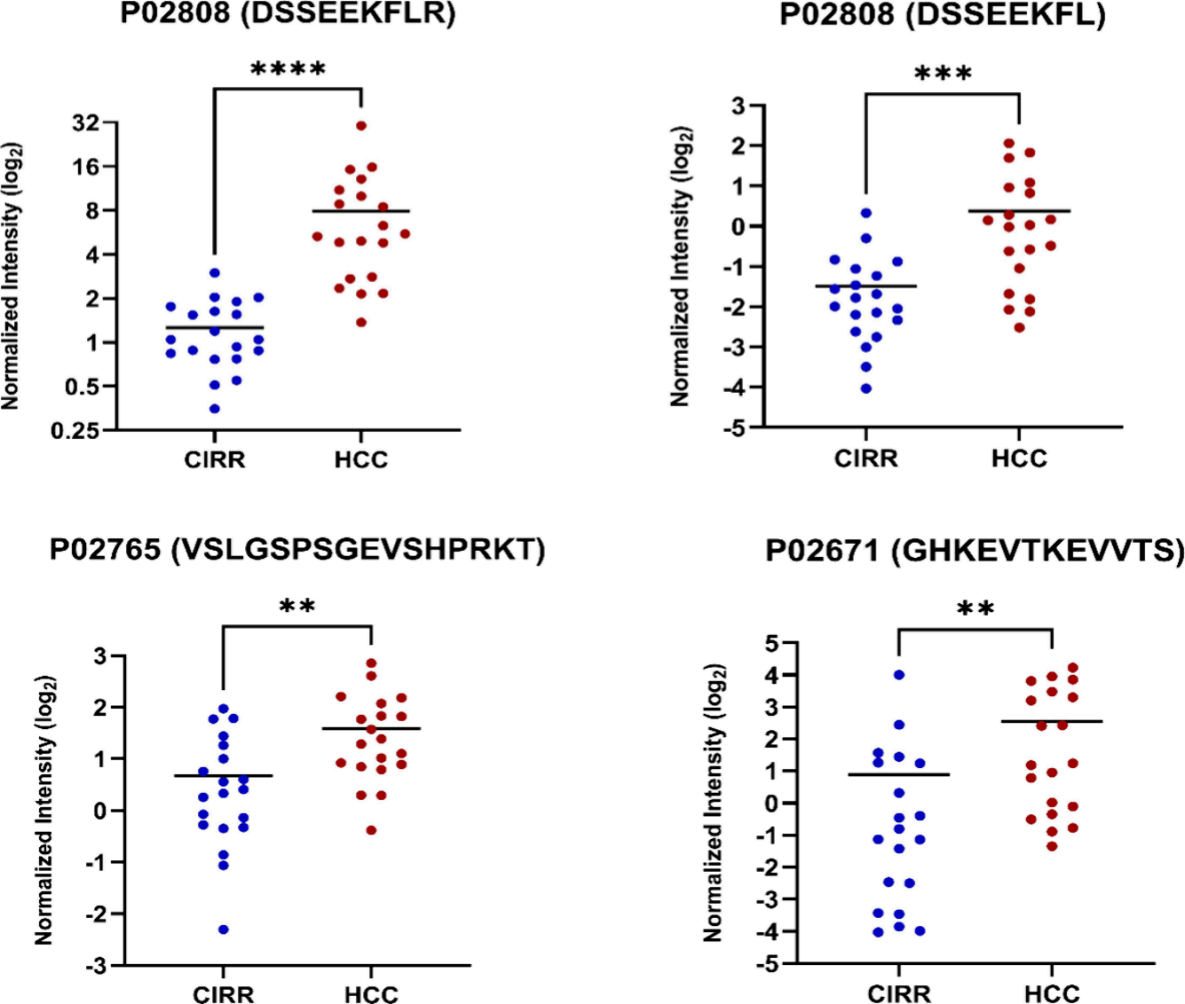
Dot plots for endogenous phosphopeptides upregulated in HCC vs CIRR (***p < 0.0001, **p < 0.001).

**Table 2:** Differentially Expressed Endogenous Phosphopeptides across HCC, CIRR, and Healthy Controls^a^

accession	protein name	gene ID	peptide sequence	*p*-value	FDR	FC
			HCC vs CIRR			
P02808	statherin	*STATH*	DSSEEKFLR	<0.0001	<0.0001	5.26
P02808	statherin	*STATH*	DSSEEKFL	0.0007	0.02415	3.16
P02765	alpha-2-HS-glycoprotein	*AHSG*	VSLGSPSGEVSHPRKT	0.0019	0.032775	2.04
P02671	fibrinogen alpha chain	*FGA*	GHKEVTKEVVTS	0.0014	0.0322	5.22
			HCC vs CTL			
P19823	interalpha-trypsin inhibitor heavy chain	*ITIH2*	SLPGESEEMMEEVD	0.0028	0.0096	2.9
P0C0L4	complement C4-A	*C4A*	YEYDELPAKDD	<0.0001	<0.0001	3.0
Q9UK05	growth/differentiation factor 2	*GDF2*	GSTEAGESSHEEDTDGHVAAGSTLA	0.0032	0.0097	1.9
P01011	alpha-1-antichymotrypsin	*SERPINA3*	NSPLDEENLTQENQDRGTHVDLG	0.0068	0.0136	–2.8
P02671	fibrinogen alpha chain	*FGA*	TFEEVSGNVSPGTRREYHTE	0.0056	0.0133	–1.8
P05546	heparin cofactor 2	*SERPIND1*	GSKGPLDQLEKGGETAQSAD	0.0047	0.0120	–2.3

a The table includes protein accession, peptide sequence, Tukey *p*-value, false discovery rate (FDR 0.05), and fold change (> ± 1.5).

Presence/absence testing by chi-squared analysis further highlighted discriminatory peptides (Table 3). PKLSPHKVQG (Q9GZP8) was uniquely detected in HCC but absent the samples from cirrhotic patients and healthy control volunteers. PRPGSTGTWNPGSSERGSAGHWTSESS and STFESKSYKMADEAGSEADHEGTH (P02671) was enriched in patients with cirrhosis but not detected in HCC cases. PLGEEDLPSEED (Q16790) and PDAKVEEEPEEEPEETA (P14625) were found over 50% HCC but absent in healthy controls. Such presence/absence patterns add another dimension to the discriminatory potential of the plasma phosphopeptidome.

**Table 3:** Statistically Differentially Expressed Endogenous Phosphopeptides Based on Chi-Squared Test of Absent and Present Calls (Adjusted *p* < 0.5)^a^

accession	protein name	gene ID	peptide sequence	HCC	CIRR	CTL
**P02671**	fibrinogen alpha chain	*FGA*	PRPGSTGTWNPGSSERGSAGHWTSESS	0	12	
			STFESKSYKMADEAGSEADHEGTH	0	9	
			MADEAGSEADHEGTHSTKRGHAKSRPV	0	3	
**Q9GZP8**	immortalization up-regulated protein	*IMUP*	PKLSPHKVQG	14	0	
**P19827**	interalpha-trypsin inhibitor heavy chain H1	*ITIH1*	SATGRSKSSEKRQAVDTAV	8	0	
**P14625**	endoplasmin	HSP90B1	PDAKVEEEPEEEPEETA	10		0
**Q16790**	carbonic anhydrase 9	*CA9*	PLGEEDLPSEED	11		0

a The numbers under the HCC, CIRR, and CTL columns indicate in how many samples the peptide was detected.

**Figure 5:**
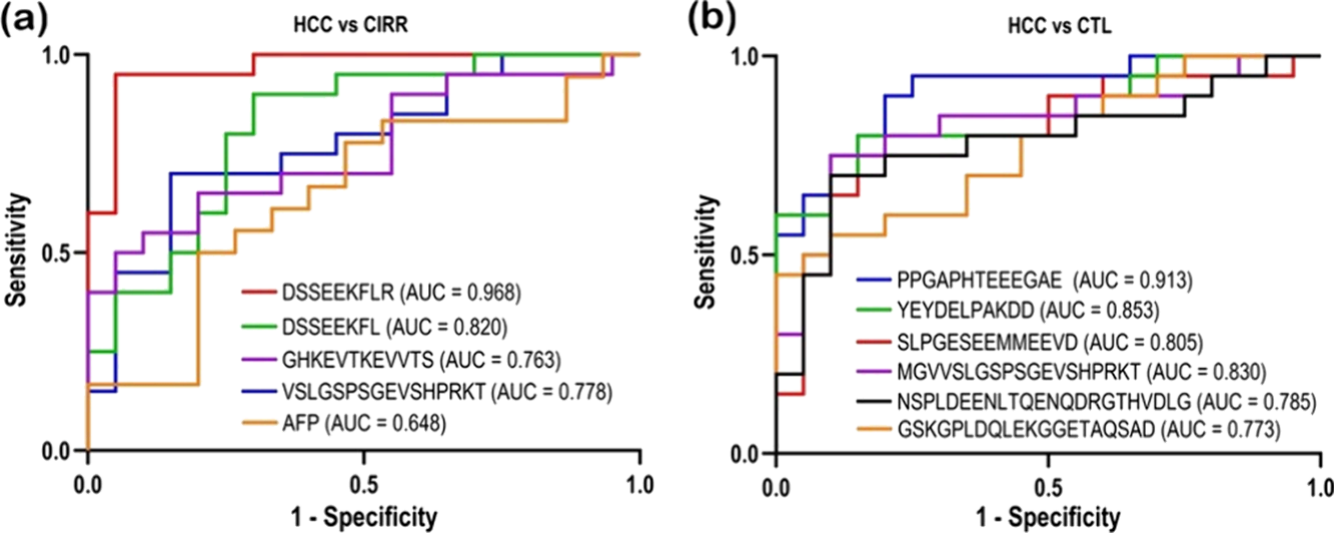
Receiver operating characteristic (ROC) curves demonstrating the diagnostic performance of the candidate biomarkers when comparing (a) HCC with CTL and (b) HCC with CIRR in comparison to AFP.

Functional enrichment analysis of the significantly altered endogenous phosphoproteins revealed strong clustering into biologically relevant categories (Figure S8). At the cellular component (GO) level, enriched terms included blood microparticles, platelet alpha granule lumen, extracellular matrix, and exosome compartments, reflecting the secretory and circulating nature of the identified phosphoproteins. Enrichment in endoplasmic reticulum and vesicle lumen further underscored the contribution of secretory pathway proteins to the circulating phosphopeptidome. At the molecular function (GO) level, the dominant terms included peptidase regulator activity, enzyme inhibitor activity, and serine-type endopeptidase inhibitor activity, consistent with the overrepresentation of serpins and protease regulatory proteins among the altered candidates. Additional enrichments in glycosaminoglycan binding, collagen binding, and extracellular matrix structural constituents highlight the functional involvement of phosphoproteins in extracellular interactions and tissue remodeling. Pathway analysis using Ingenuity Pathway Analysis (IPA) confirmed activation of several disease-relevant signaling cascades. The top canonical pathways included coagulation system, integrin signaling, LXR/RXR activation, acute-phase response signaling, and post-translational protein phosphorylation (Figure 6). Additional pathways such as syndecan interactions, integrin–cell surface interactions, and calcium-mediated platelet activation were also significantly represented, suggesting links to vascular remodeling and immune modulation. The protein–protein interaction (PPI) network analysis highlighted tightly connected modules formed by apolipoproteins (APOA1, APOC3), complement factors (C4A), serpins (SERPINA3, ITIH2), and structural proteins (FN1, SPARC, collagens). These clusters converge on key regulators such as integrins, TGF-β, and lipid metabolism proteins, with LXR/RXR emerging as a central hub. The network topology underscores the coordinated regulation of lipid transport, coagulation, and immune signaling, all of which are processes strongly implicated in hepatocellular carcinoma pathogenesis.

**Figure 6:**
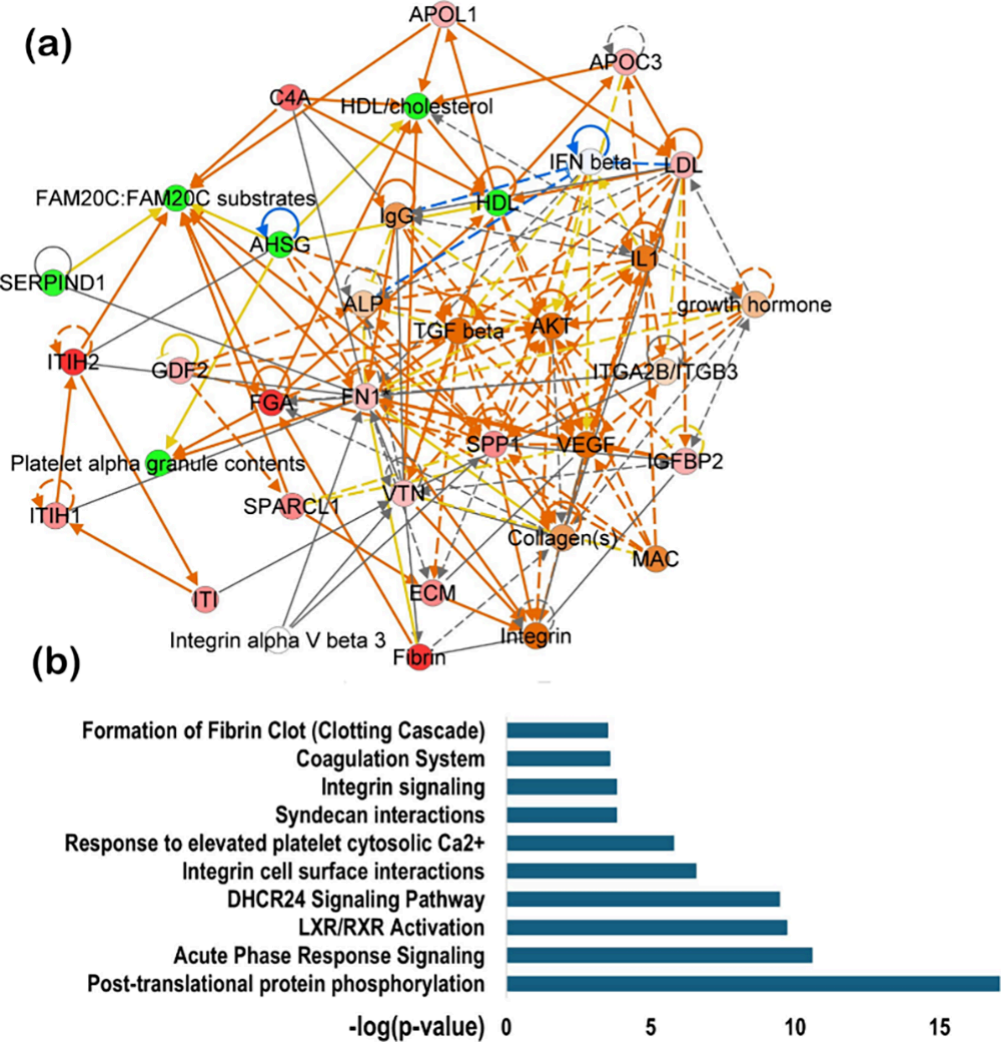
Ingenuity pathway analysis (IPA). (a) Protein–protein interaction (PPI) network (b) top 10 canonical pathway enriched of differentially expressed precursor proteins.

### Diagnostic Performance

To evaluate the diagnostic potential of the identified endogenous phosphopeptides, receiver operating characteristic (ROC) curve analysis was performed. In the HCC vs CTL comparison, several peptides demonstrated strong discriminatory power shown in Figure 5b. The peptide PPGAPHTEEEGAE exhibited the highest diagnostic accuracy with an AUC of 0.913, indicating excellent sensitivity and specificity. Other peptides, including YEYDELPAKDD (AUC = 0.853), MGVVSLGSPSGEVSHPRKT (AUC = 0.830), and SLPGESEEMMEEVD (AUC = 0.805), also showed robust classification performance. Additional candidates such as NSPLDEENLTQENQDRGTHVDLG (AUC = 0.785) and GSKGPLDQLEKGGETAQSAD (AUC = 0.773) demonstrated moderate but consistent diagnostic potential. In the HCC vs CIRR comparison shown in Figure 5a, the peptide DSSEEKFLR achieved the highest diagnostic performance with an AUC of 0.968, reflecting excellent discrimination between the two groups. Its truncated variant DSSEEKFL also performed well with an AUC of 0.820. Other peptides, including VSLGSPSGEVSHPRKT (AUC = 0.778) and GHKEVTKEVVTS (AUC = 0.763), showed moderate diagnostic accuracy. By contrast, alpha-fetoprotein (AFP), based on clinical serum data rather than MS analysis, yielded only modest discrimination with an AUC of 0.648, performing considerably worse than the MS-derived endogenous phosphopeptides.

## Discussion

This study represents, to our knowledge, the first comprehensive characterization of the plasma endogenous phosphopeptidome in HCC, benchmarked against CIRR and CTL. By applying a digestion-free workflow with enrichment of phosphorylated peptides, we captured 1,365 unique endogenous phosphopeptides corresponding to 549 precursor proteins. The high reproducibility of our data set, reflected in strong correlations across 60 plasma samples (*r* > 0.9) and consistent HeLa QC performance, validates the robustness of this approach for studying phosphorylation-dependent plasma signatures. The observation that PLS-DA separated the disease groups from healthy controls with minimal overlap demonstrates that the circulating phosphopeptidome contains strong disease-specific information. These findings extend earlier serum and plasma proteomics studies that focused on the digested proteome rather than the endogenous peptidome. Villanueva et al. reported large-scale proteomic alterations in HCC serum, identifying apolipoproteins and complement proteins as candidate biomarkers,^27^ while Kimhofer et al. highlighted proteomic and metabolomic markers linked to coagulation and immune signaling.^28^ Our study builds on this knowledge by demonstrating that phosphorylation-specific endogenous peptides, which represent direct in vivo proteolytic and signaling states, can differentiate HCC from both CIRR and CTL individuals. Complement C4-A (YEYDELPAKDD) and Interalpha-trypsin inhibitor heavy chain H4 (SLPGESEEMMEEVD) were markedly elevated,^29^ consistent with activation of complement and acute-phase response pathways in HCC plasma.^30^ In contrast, peptides from Alpha-1-antichymotrypsin (NSPLDEENLTQENQDRGTHVDLG), Heparin cofactor II (GSKGPLDQLEKGGETAQSAD), and Fibrinogen alpha chain (TFEEVSGNVSPGTRREYHTE**)** were reduced, indicating impaired protease inhibition and anticoagulant regulation. Together, these shifts point to coordinated dysregulation of complement coagulation and acute-phase signaling, highlighting the plasma endogenous phosphopeptidome as a sensitive indicator of early hepatocellular carcinoma–associated biochemical changes. The HCC vs CIRR comparison highlighted statherin-derived peptides (*DSSEEKFLR* and *DSSEEKFL*) are highly discriminatory. Statherin, a phosphorylated protein, is classically associated with saliva, but its presence in plasma at a very low level^31,32^ and strong upregulation in HCC is novel, suggesting an unexplored role in systemic signaling or tumor biology. The parallel increase of Fibrinogen alpha chain (GHKEVTKEVVTS) and Alpha-2-HS-glycoprotein (VSLGSPSGEVSHPRKT) further supports activation of the coagulation and acute-phase response systems, both of which are tightly linked to hepatic inflammation, matrix remodeling, and tumor progression. These findings align with prior observations that fibrinogen and fetuin-A (AHSG) undergo extensive phosphorylation during chronic liver injury, modulating cell adhesion and signaling cascades associated with oncogenic transformation.

The distinct presence–absence patterns of endogenous phosphopeptides across disease states likely reflect underlying alterations in protease activity, extracellular matrix turnover, and phosphorylation-driven signaling during liver disease progression. The exclusive appearance of PKLSPHKVQG in HCC plasma suggests tumor-specific proteolytic processing or kinase-mediated modification events that generate unique circulating fragments absent in cirrhotic or healthy control states. Conversely, the disappearance of fibrinogen-derived peptides such as PRPGSTGTWNPGSSERGSAGHWTSESS and STFESKSYKMADEAGSEADHEGTH in HCC compared with cirrhosis may indicate progressive degradation or altered secretion of coagulation-related proteins as fibrosis transitions to malignancy. Together, these qualitative shifts in phosphopeptide occurrence highlight that the circulating phosphopeptidome not only encodes quantitative abundance changes but also captures dynamic molecular turnover events, offering mechanistic insight into tumor-associated protease dysregulation and matrix remodeling in HCC. Such binary patterns have been used in other body-fluid proteomics studies as robust diagnostic indicators, particularly when quantitative fold changes may be subtle.^33^

The ROC analyses provide strong translational support for these observations. Individual peptides demonstrated diagnostic accuracies that substantially outperformed AFP, the current clinical standard. In particular, PPGAPHTEEEGAE discriminated HCC from healthy controls with an AUC of 0.913, while statherin-derived DSSEEKFLR distinguished HCC from cirrhosis with an AUC of 0.968. These values exceed the typical performance of AFP (AUC 0.6–0.7 in most clinical studies), reinforcing the potential of endogenous phosphopeptides as superior biomarkers. Importantly, the top-performing peptides mapped back to biological pathways identified in enrichment analyses, further validating their mechanistic plausibility. For example, fibrinogen- and alpha-1-antichymotrypsin–derived peptides link directly to coagulation and acute-phase pathways, while ApoA1- and statherin-derived peptides align with lipid metabolism and extracellular remodeling.

The functional enrichment and pathway analysis of differentially expressed endogenous phosphoproteins revealed that HCC progression is strongly reflected in the circulating phosphopeptidome. GO enrichment at the cellular component level highlighted extracellular compartments including blood microparticles, platelet alpha granule lumen, and exosomes. This finding underscores the role of secretory and vesicle-associated proteins in shaping the plasma phosphopeptidome, consistent with recent reports that extracellular vesicles serve as important carriers of post-translationally modified peptides in cancer and liver disease.^34,35^ At the molecular function level, the enrichment of peptidase regulator activity and serine-type endopeptidase inhibitor activity points to a central role of protease–antiprotease balance in the systemic circulation of HCC patients. The predominance of serpins and related protease inhibitors in our data set aligns with previous proteomics studies linking dysregulated protease activity to liver injury, tissue remodeling, and tumor microenvironment dynamics.^36^ The additional enrichment of extracellular matrix and glycosaminoglycan binding functions further indicates that circulating phosphoproteins are closely tied to processes of matrix turnover and fibrosis, both of which are fundamental to cirrhosis and HCC development. Ingenuity Pathway Analysis (IPA) provided deeper insight into disease-relevant signaling networks. The coagulation system and acute-phase response signaling emerged among the most significant pathways, highlighting systemic inflammation and hemostatic imbalance as key features of HCC biology. This is consistent with earlier plasma proteomics and clinical data showing that hypercoagulability and inflammatory mediators contribute to both cirrhosis progression and hepatocarcinogenesis.^37^ Importantly, the enrichment of the LXR/RXR axis suggests altered lipid metabolism and nuclear receptor signaling in the plasma phosphopeptidome of HCC patients. LXR/RXR signaling has been previously implicated in hepatic lipid regulation, immune modulation, and tumor-promoting processes,^38^ and our findings provide phosphopeptide-level evidence of its systemic dysregulation in HCC. The protein–protein interaction (PPI) network analysis supports these observations, revealing highly connected clusters composed of apolipoproteins (APOA1, APOC3), complement components (C4A), serpins (SERPINA3, ITIH2), and extracellular matrix proteins (FN1, SPARC, collagens). The network topology emphasizes the interplay between lipid transport, complement activation, and extracellular remodeling, with integrins and TGF-β signaling nodes acting as central regulators. These hubs are known to drive cell adhesion, migration, and immune interactions in the tumor microenvironment.^39^ Together, the enrichment and network analyses demonstrate that the altered plasma phosphopeptidome in HCC captures a convergent signature of inflammation, coagulation, matrix remodeling, and lipid signaling. Overall, this systems-level view extends beyond individual peptide markers, revealing that endogenous phosphopeptides map onto coordinated biological processes relevant to HCC pathogenesis. These results suggest that the plasma phosphopeptidome may serve not only as a diagnostic biomarker reservoir but also as a window into the systemic signaling changes that accompany liver cancer progression. This study has several strengths, including the digestion-free workflow that preserved in vivo phosphorylation, rigorous QC to ensure reproducibility, and the inclusion of cirrhosis controls, which addresses the critical diagnostic challenge of distinguishing HCC from cirrhosis. Limitations include the modest cohort size and the absence of validation in an independent population. Future studies should validate these candidate peptides using targeted assays such as PRM or MRM in larger cohorts, ideally including early stage HCC patients.

## Conclusions

This study provides the first in-depth characterization of the plasma endogenous phosphopeptidome in hepatocellular carcinoma (HCC), revealing its strong potential as a minimally invasive source of circulating biomarkers. Using high-resolution LC–MS/MS and integrative statistical analysis, we identified several phosphopeptides with outstanding diagnostic performance. The statherin-derived peptide DSSEEKFLR achieved an AUC = 0.968 for distinguishing HCC from cirrhosis far exceeding AFP (AUC = 0.648) while its truncated variant DSSEEKFL (AUC = 0.820) reinforces the findings. Additional candidates, including PPGAPHTEEEGAE (NST1), YEYDELPAKDD (C4A), SLPGESEEMMEEVD (ITIH4), and VSLGSPSGEVSHPRKT (AHSG), also displayed high accuracy (AUC > 0.80). Together, these findings highlight a novel phosphopeptide signature that captures disease-specific proteolytic and phosphorylation events, offering a promising foundation for blood-based diagnostics and clinical monitoring of HCC. While these findings are promising, validation of the candidate biomarkers via absolute quantification in a larger sample size is needed. Future work will focus on validating these candidates using targeted assays and assessing their performance across broader clinical populations.

## Supplementary Material





## Data Availability

Data generated in this work is available via the ProtemeXchange Consortium via the PRIDE partner repository with the identifier PXD070164.

## Notes

(Figure S1) Correlation among the human plasma profiling from 60 subjects; (Figure S2) physicochemical properties of identified endogenous phosphoproteins and phosphopeptides; (Figure S3) physicochemical properties of identified endogenous phosphoproteins and phosphopeptides; (Figure S4) group abundance distribution of phosphopeptide across three groups; (Figure S5) group abundance distribution of phosphoproteins across three groups; (Figure S6) distribution of log-transformed proteins abundances from QC (HeLa digest) during the MS data accusation for three groups; (Figure S7) heatmap of the 69 endogenous phosphopeptides significantly altered across three groups by one-way ANOVA (FDR *q* < 0.05); (Figure S8) functional enrichment analysis; (Table S1) differentially expressed endogenous phosphopeptides identified by ANOVA across HCC, cirrhosis, and healthy controls (PDF)
(Table S2) Endogenous phosphopeptides detected (XLSX)
