# Supplementary material for: Phosphopeptidome Profiling of Human Plasma for Hepatocellular Carcinoma Biomarker Discovery
Source: J Proteome Res. 2025 Dec 31;25(2):1115–25. doi: 10.1021/acs.jproteome.5c01004 (PMC12831616; doi:10.1021/acs.jproteome.5c01004)
Supplement: Supplementary file 1 [file pr5c01004_si_001.pdf]

Phosphopeptidome Profiling of Human Plasma for Hepatocellular Carcinoma Biomarker Discovery

Shafaq Saleem[1], Muhammad Salman Sajid[1], Rency S. Varghese[1], Zaki A. Sherif[2], Alexander Kroemer[3], Habtom W. Ressom[1*]

[1]Department of Oncology, Lombardi Comprehensive Cancer Center, Georgetown University Medical Center, Washington, DC 20057, USA

[2]Department of Biochemistry & Molecular Biology, Howard University College of Medicine, Washington, DC 20059, USA

[3]MedStar Georgetown Transplant Institute, MedStar Georgetown University Hospital and the Center for Translational Transplant Medicine, Georgetown University Medical Center, Washington, DC, 20057, USA.

*Corresponding Author
Prof. Habtom W. Ressom
Department of Oncology
Lombardi Comprehensive Cancer Center
Georgetown University Medical Center
Washington, DC 20057, USA
Email: hwr@georgetown.edu

Table of Content

List of Abbreviations S3

Figure S1: Correlation among the human plasma profiling. S5

Figure S2: Physicochemical properties S6

Figure S3: Physicochemical properties S7

Figure S4: Group abundance distribution of phosphopeptides across three groups. S8

Figure S5: Group abundance distribution of phosphoproteins across three groups. S8

Figure S6: Protein abundances from QC (HeLa digest) S9

Figure S7: Heatmap of the **69** endogenous phosphopeptides significantly S10

Figure S8: Functional enrichment analysis S11

Table S1: Differentially expressed endogenous phosphopeptides identified by ANOVA S12

Table S2: Endogenous phosphopeptides detected (XLSX)

Abbreviation	*Full Term*
AA	African American
Ab+ / Ag+	Antibody positive / antigen positive (serology)
ACN	Acetonitrile
AFP	Alpha-fetoprotein
ANOVA	Analysis of variance
BP	Biological process
CC	Cellular component
CIRR	Cirrhosis
CTL	Healthy controls
DDA	Data-dependent acquisition
EA	European American
FA	Formic acid
FDR	False discovery rate
GO	Gene Ontology
HCD	Higher-energy collisional dissociation
HCC	Hepatocellular carcinoma
HIPAA	Health Insurance Portability and Accountability Act
IPA	Ingenuity Pathway Analysis
IRB	Institutional Review Board
KNN	K-nearest neighbors (imputation)
LC–MS/MS	Liquid chromatography–tandem mass spectrometry
LFQ	Label-free quantification
LXR/RXR	Liver X receptor/Retinoid X receptor
m/z	Mass-to-charge ratio
MF	Molecular function

MRM	Multiple reaction monitoring
MWCO	Molecular weight cutoff
nano-LC	Nano-flow liquid chromatography
NCE	Normalized collision energy
PCA	Principal component analysis
PLS-DA	Partial least squares–discriminant analysis
PPI	Protein–protein interaction
PRM	Parallel reaction monitoring
QC	Quality control
TFA	Trifluoroacetic acid
TNM	Tumor-node-metastasis staging

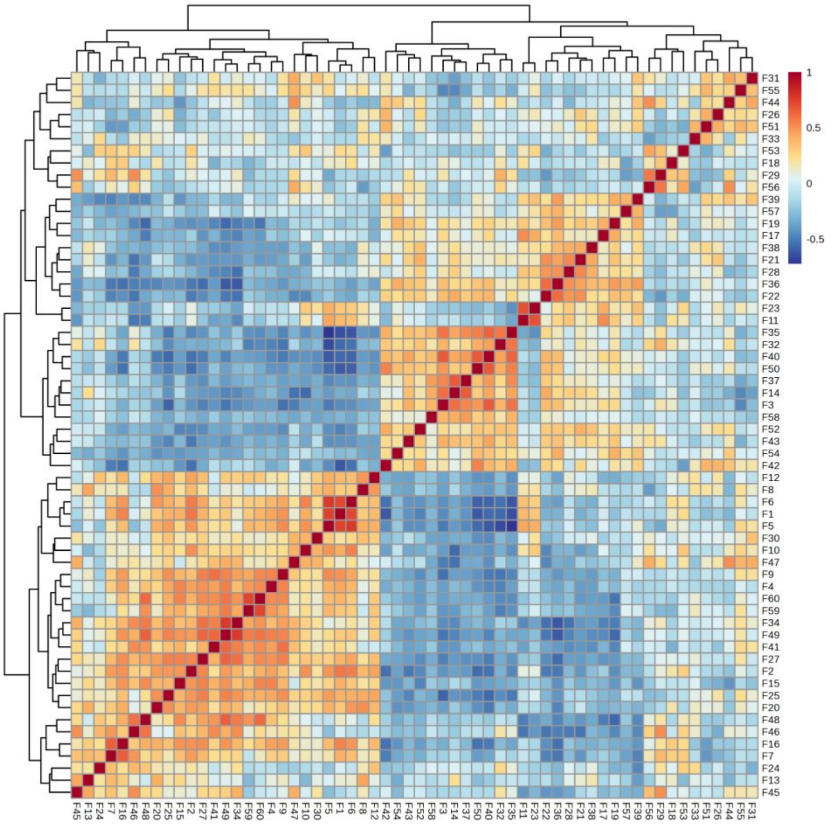

Figure S1: Correlation among the human plasma profiling from 60 subjects.

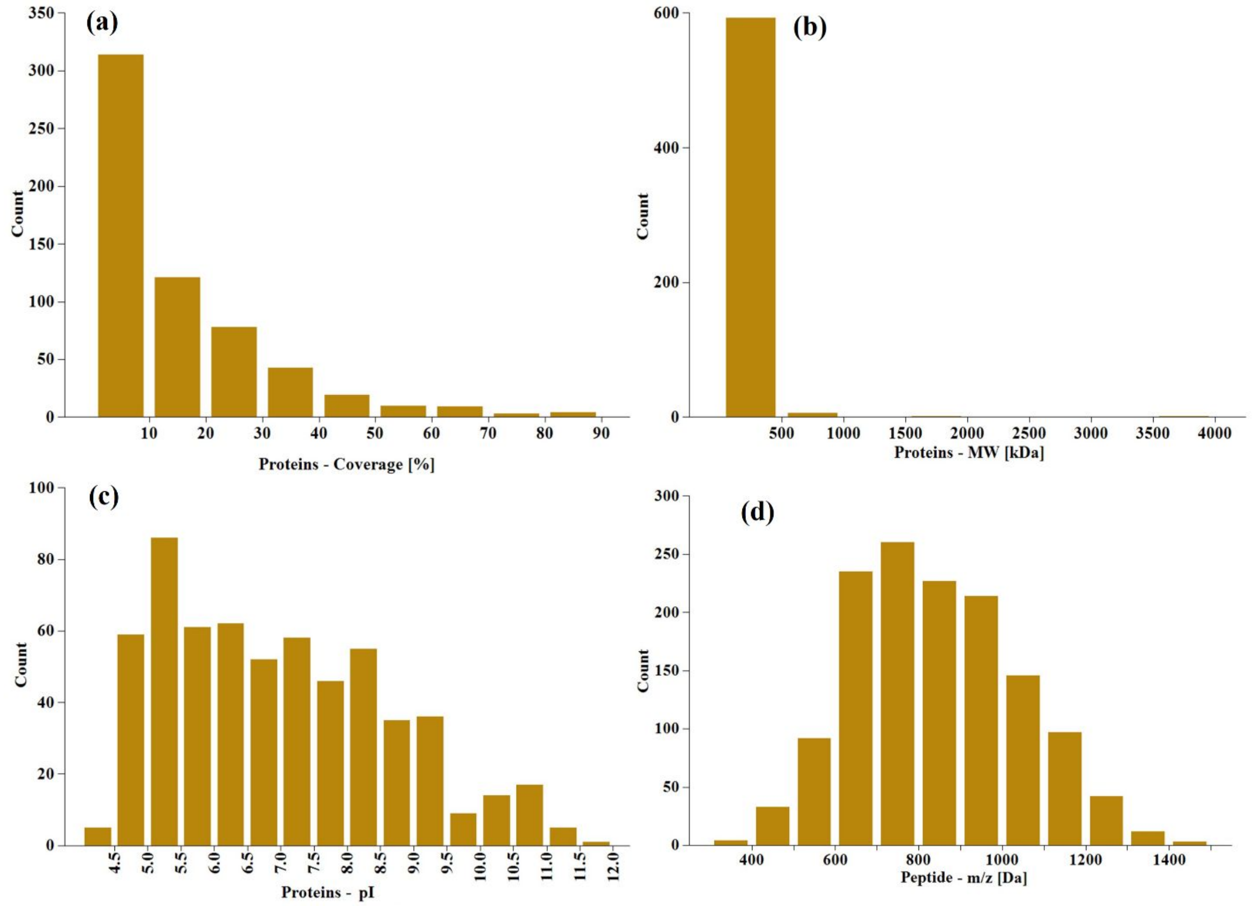

Figure S2. Physicochemical properties of identified endogenous phosphoproteins and phosphopeptides. (a) Distribution of protein sequence coverage (b) Molecular weight (MW) distribution of identified proteins (c) Isoelectric point (pI) distribution of proteins (d) Mass-to-charge (m/z) distribution of identified phosphopeptides.

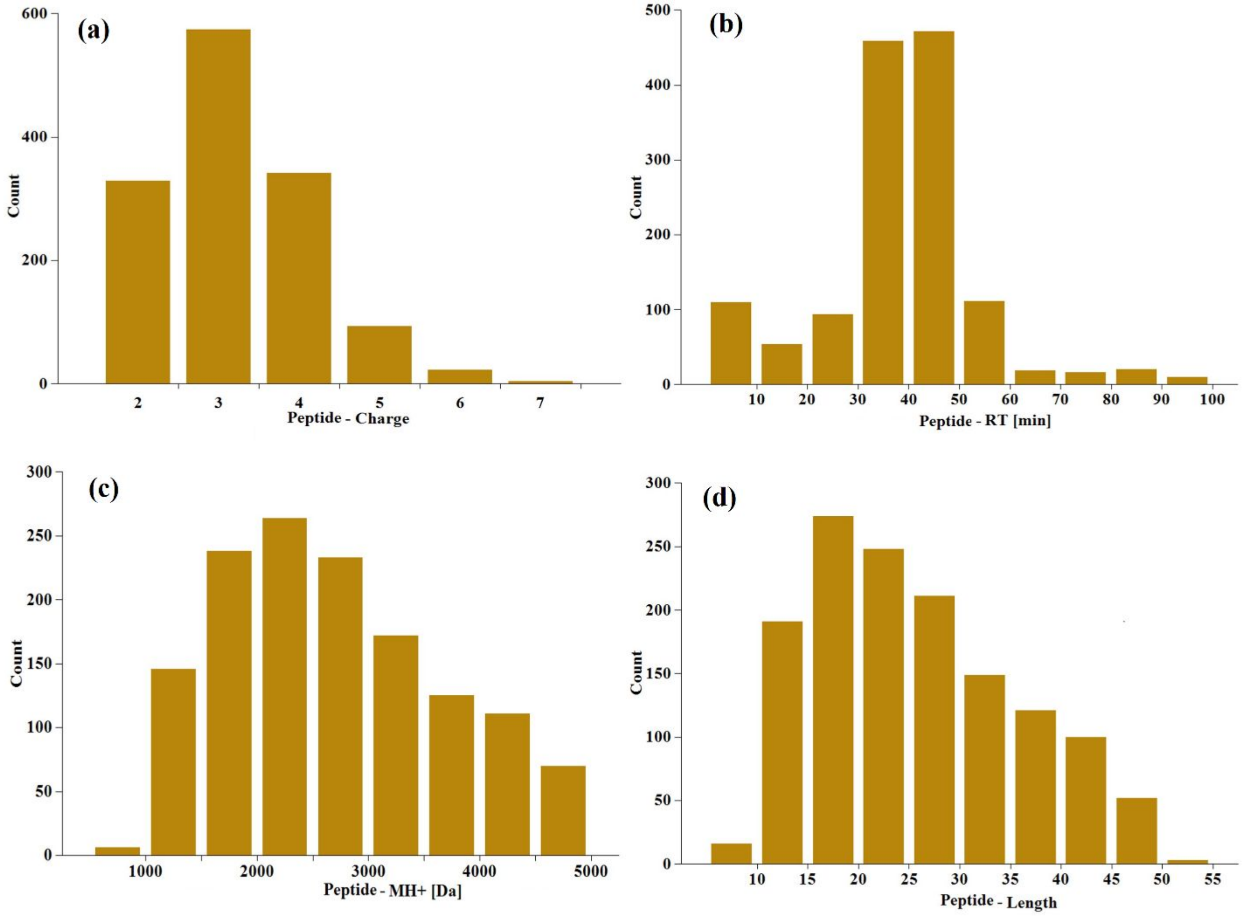

Figure S3. Physicochemical properties of identified endogenous phosphoproteins and phosphopeptides**.** (a) Distribution of peptide charge (b) Retention time (RT) distribution of identified phosphopeptides (c) phosphopeptides MH^+ distribution (d) peptides length distribution of identified phosphopeptides.

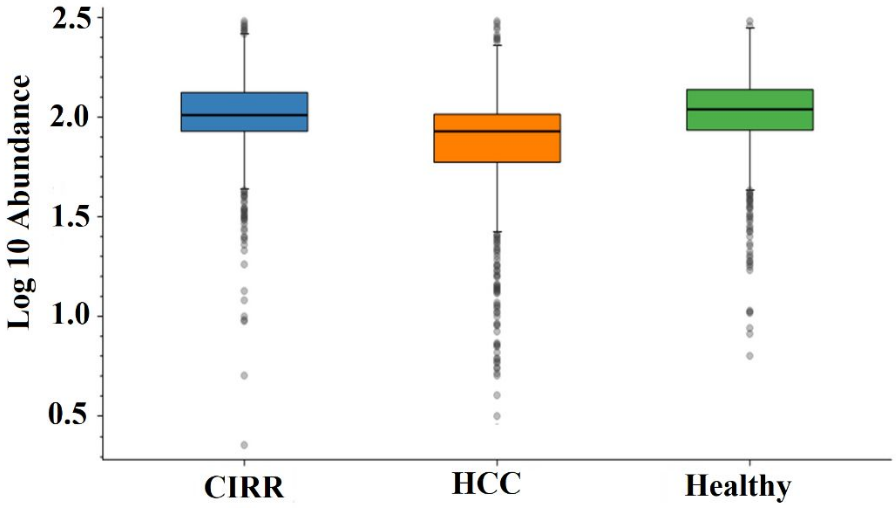

Figure S4. Group abundance distribution of phosphopeptide across three groups.

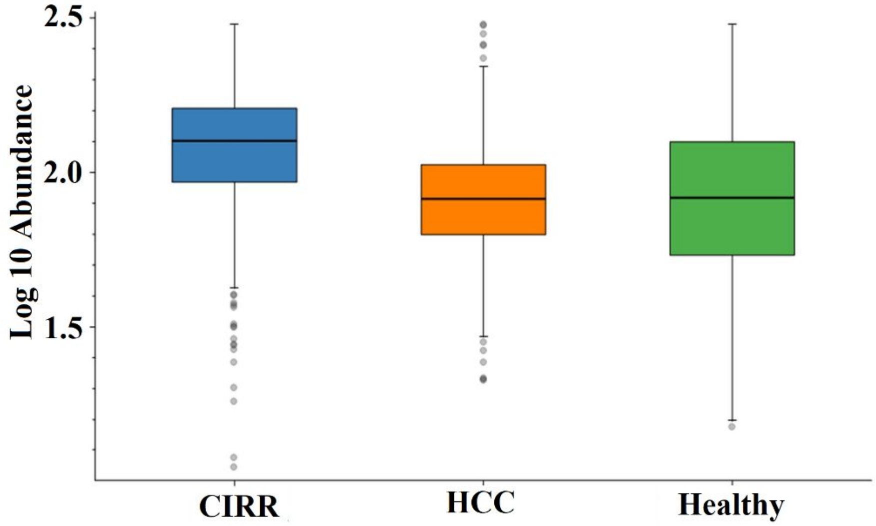

Figure S5. Group abundance distribution of phosphoproteins across three groups.

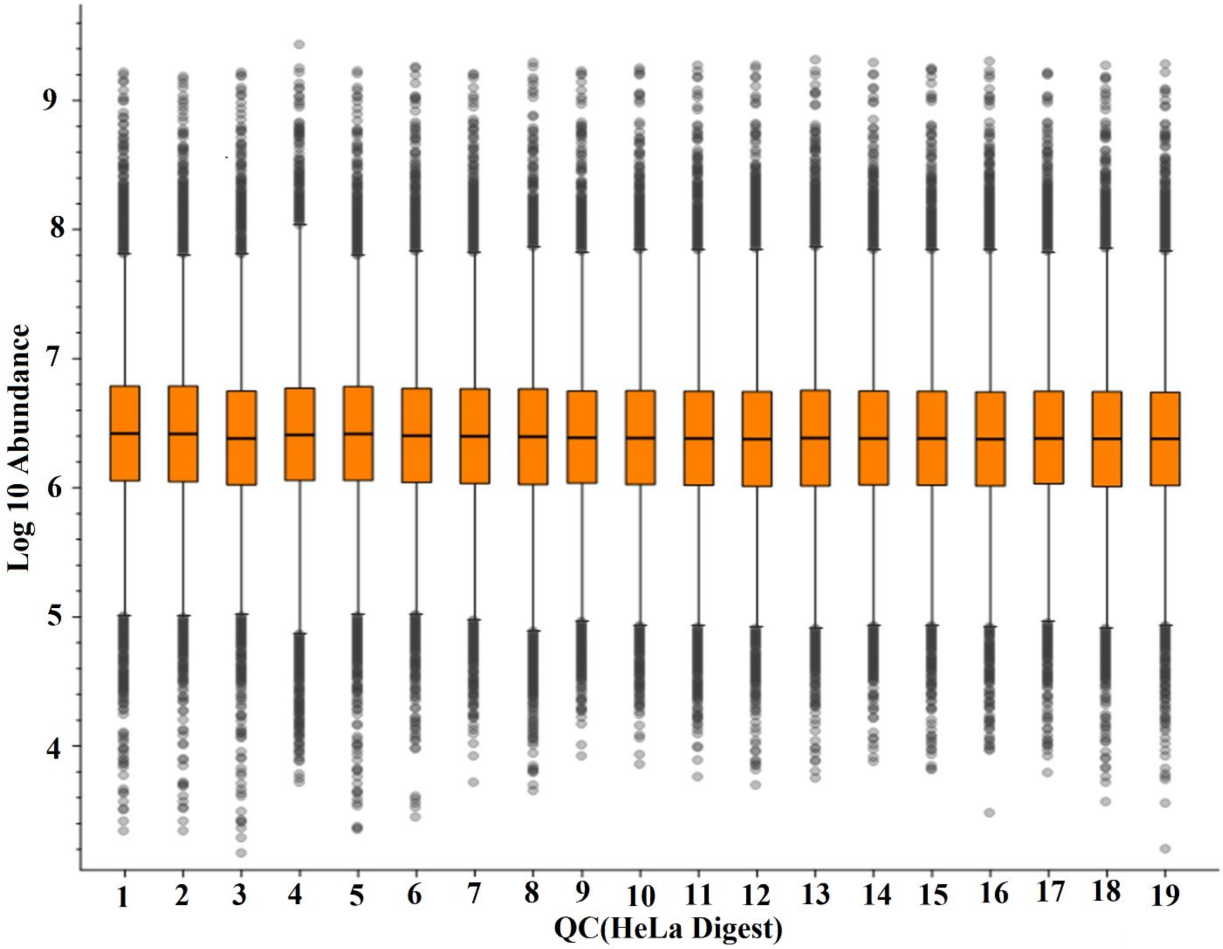

Figure S6. Distribution of log-transformed proteins abundances from QC (HeLa digest) during the MS data accusation for three groups.

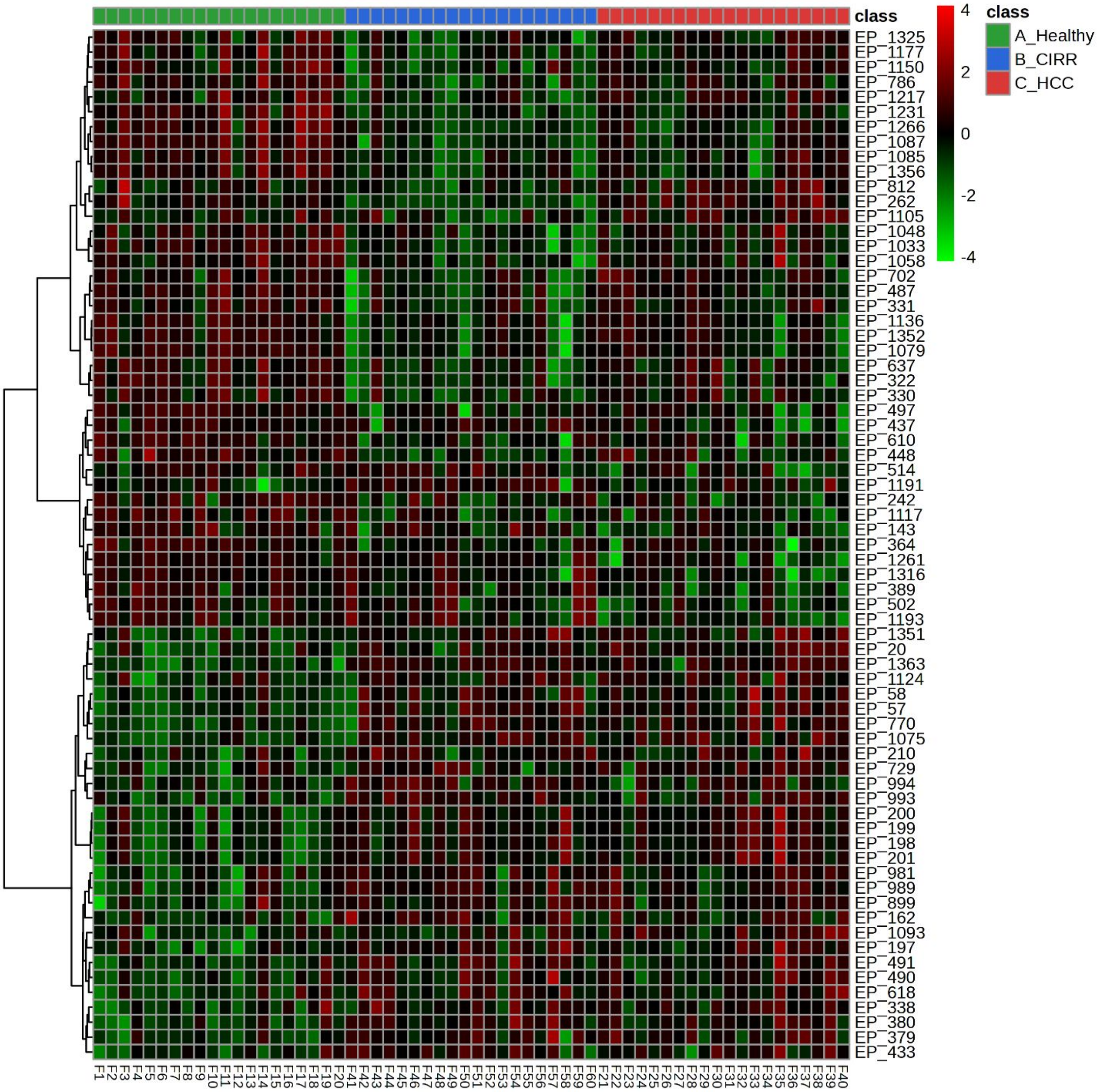

Figure S7: Heatmap of the 69 endogenous phosphopeptides significantly altered across CTL (n=20), CIRR (n=20), and HCC (n=20) by one-way ANOVA (FDR q<0.05).

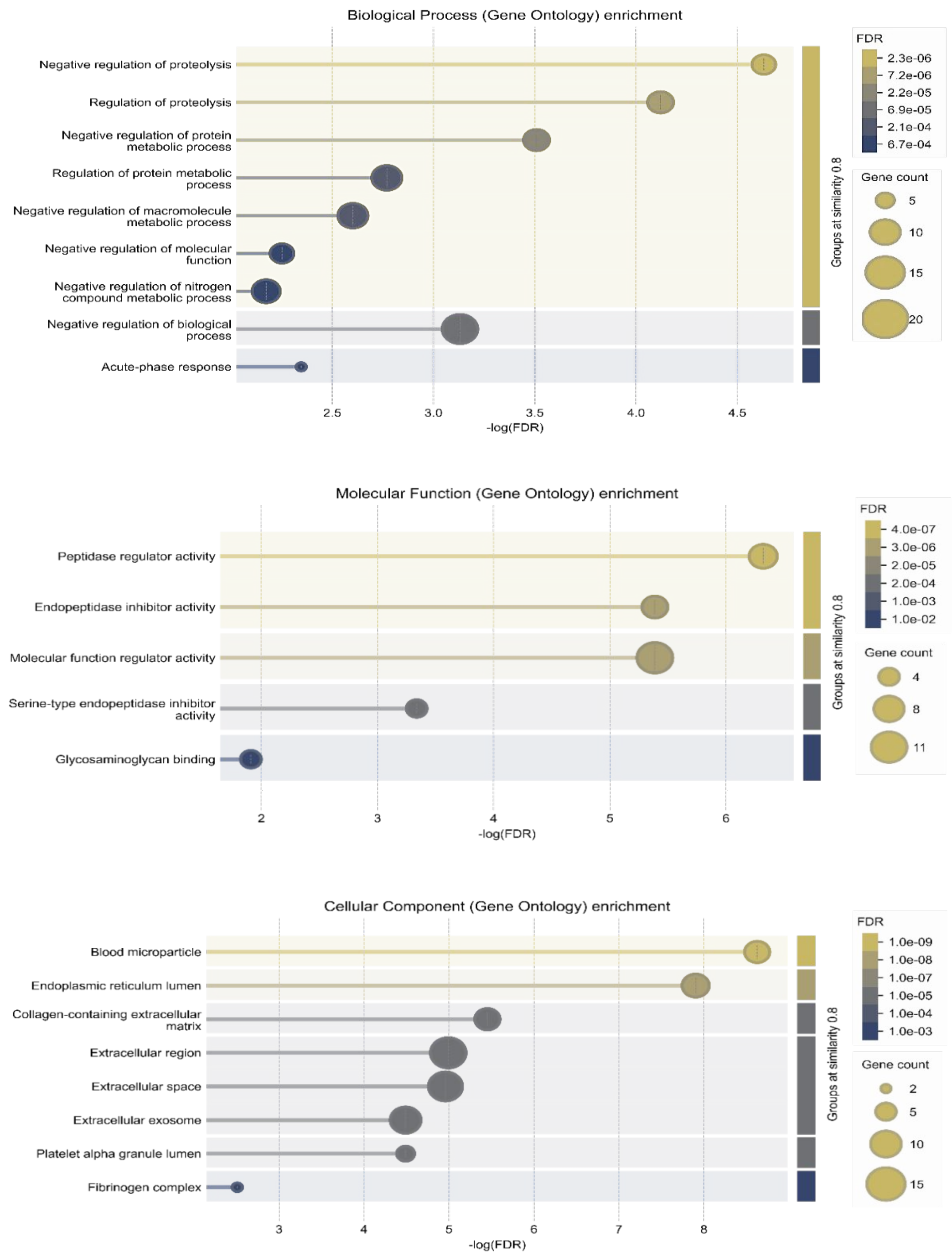

Figure S8: Functional enrichment analysis. Gene ontology (GO) enrichment analysis highlighting biological processes (BP), molecular functions (MF), and cellular components (CC) associated with DEPs.

Table S1. Differentially expressed endogenous phosphopeptides identified by ANOVA across HCC, cirrhosis, and healthy controls. The table includes protein accession, peptide sequence, ANOVA p-value, false discovery rate (FDR), and associated statistical significance.

Master Protein	*Peptide Sequence*	*ANOVA p_Value*	*ANOVA FDR*
P02671	STFESKSYKMAD	1.91E-09	4.03E-07
P02808	DSSEEKFLR	1.79E-09	4.03E-07
P02671	STFESKSYKM	3.72E-08	5.22E-06
P19827	SKSSEKRQAVDTAVD	1.26E-07	1.06E-05
P02751	MPLDVQADREDSRE	1.15E-07	1.06E-05
Q14515	PNISNGEEEEEKEPGEVGTHND	1.82E-06	0.00011
P02671	STFESKSY	1.63E-06	0.00011
P41271	PPGAPHTEEEGAE	3.39E-06	0.000178
P10451	VKQADSGSSEEKQLYNKYPD	1.13E-05	0.00053
P02765	SLGSPSGEVSHPRKT	1.87E-05	0.000789
P01011	HPNSPLDEENLTQ	3.54E-05	0.001064
P02671	PGSPRPGSTGTWNPGSSE	3.46E-05	0.001064
P02765	MGVVSLGSPSGEVSHPRKT	3.02E-05	0.001064
O14791	PQSKPLGDWAAGTMD	3.04E-05	0.001064
P01011	HPNSPLDEENLTQE	5.36E-05	0.001504
P0C0L4	YEYDELPAKDD	7.42E-05	0.001951
P01011	HPNSPLDEENLTQENQ	7.98E-05	0.001976
P02671	STFESKSYKMA	8.83E-05	0.002065
P02671	STFESKSYKMADEAGSEA	0.00011	0.002307
P02671	DSTFESKSYKMA	0.000105	0.002307
P02765	GVVSLGSPSGEVSHPR	0.000242	0.004855
P02671	PGSPRPGSTGTWNPGS	0.000291	0.005441
P02765	VVSLGSPSGEVSHPR	0.000297	0.005441
P02765	VVSLGSPSGEVSHPRKT	0.000383	0.006726
P02656	PEVRPTSAVAA	0.000409	0.006894
P02790	LPPTSAHGNVAEGETKPD	0.000593	0.009599
P02765	VSLGSPSGEVSHPRKT	0.000661	0.01031
P01011	HPNSPLDEENLTQEN	0.00074	0.010855
P02671	PGSPRPGSTGTWNPGSS	0.000748	0.010855
P01011	HPNSPLDEENLT	0.000786	0.011036
P02671	SSGTGSTGNQNPGSPRPGSTGTWNPGSSERGSAGHWTSESSVSG	0.000904	0.011536
P05546	GSKGPLDQLEKGGETAQSAD	0.0009	0.011536
P02765	LGSPSGEVSHPR	0.000863	0.011536
P02808	DSSEEKFL	0.001053	0.013042
P02671	PRPGSTGTWNPGSSERGSAG	0.00119	0.014309
P02751	LDVQADREDSRE	0.001276	0.014922
P41271	PPGAPHTEEEGAED	0.001315	0.014963
P02671	PGSSERGSAGHWTSESSVSGSTGQWH	0.001508	0.016707
P02671	QNPGSPRPGSTGTWNPGSSERG	0.001712	0.018484

P02671	SPRPGSTGTWNPGSSERGSAGH	0.001825	0.019206
P02671	PGSPRPGSTGTWNPGSSERGSAGHWTSESS	0.001994	0.019984
P02671	NPGSPRPGSTGTWNPGSSE	0.001971	0.019984
P02671	GHKEVTKEVVTS	0.002094	0.020498
P19827	SATGRSKSSEKRQAVDTAVD	0.002224	0.021279
P02765	GVVSLGSPSGEVSHPRKT	0.00262	0.024512
P02671	VTKEVVTSEDGSD	0.002729	0.024977
P02671	SPRPGSTGTWNPGSS	0.003057	0.027381
P19823	SLPGESEEMMEEVD	0.003158	0.027697
P02671	SPRPGSTGTWNPGSSE	0.003438	0.029541
P02671	SPRPGSTGTWNPGSSERGSAGHWTSESSVSGSTG	0.003783	0.031853
P02671	NQNPGSPRPGSTGTWNPGSSERGSAGH	0.003954	0.032641
Q9UK05	GSTEAGESSHEEDTDGHVAAGSTLA	0.004053	0.032813
P21675	DIPSATPEKQVTQEGEDG	0.004229	0.03345
P02751	PLDVQADREDSRE	0.00429	0.03345
Q92954	TSKETSLTVNKE	0.004543	0.034777
P02671	TGSTGNQNPGSPRPGSTGTWNPGSSERGSAGHWTSESSVSG	0.004722	0.034887
P02671	STFESKSYKMADEAGSEAD	0.004723	0.034887
P02671	TWNPGSSERGS	0.005037	0.036563
P02765	LGSPSGEVSHPRKT	0.005208	0.037165
P02671	TGSTGNQNPGSPRPGSTGTWNPGS	0.005519	0.038725
P02671	TGSTGNQNPGSPRPGSTGTWNPGSSERGSAGH	0.006469	0.044647
P02671	NARPNNPDWGTFE	0.006611	0.044674
P02671	PRPGSTGTWNPGSSE	0.006685	0.044674
P01011	NSPLDEENLTQENQDRGTHVDLG	0.007179	0.047223
P02671	TFEEVSGNVSPGTRREYHTE	0.007395	0.047896
P02671	PGSSERGSAGHWTSESSVSGSTG	0.007538	0.048086
P02671	SSERGSAGHWTSES	0.007728	0.048562
P02671	NPGSPRPGSTGTWNPGS	0.008079	0.049881
P02671	SPGSGNARPNNPDWGT	0.008175	0.049881
